# Quantitative Trait Locus Analysis of Seed Germination and Seedling Vigor in *Brassica rapa* Reveals QTL Hotspots and Epistatic Interactions

**DOI:** 10.3389/fpls.2015.01032

**Published:** 2015-12-01

**Authors:** Ram K. Basnet, Anita Duwal, Dev N. Tiwari, Dong Xiao, Sokrat Monakhos, Johan Bucher, Richard G. F. Visser, Steven P. C. Groot, Guusje Bonnema, Chris Maliepaard

**Affiliations:** ^1^Wageningen UR Plant Breeding, Wageningen University and Research Center, Wageningen UniversityWageningen, Netherlands; ^2^Centre for BioSystems GenomicsWageningen, Netherlands; ^3^State Key Laboratory of Crop Genetics and Germplasm Enhancement, Horticultural College, Nanjing Agricultural UniversityNanjing, China; ^4^Russian State Agrarian University, Moscow Timiryazev Agricultural AcademyMoscow, Russia; ^5^Plant Research InternationalWageningen, Netherlands

**Keywords:** *Brassica rapa*, QTL mapping, seed germination, seedling vigor, salt stress, candidate genes

## Abstract

The genetic basis of seed germination and seedling vigor is largely unknown in *Brassica* species. We performed a study to evaluate the genetic basis of these important traits in a *B. rapa* doubled haploid population from a cross of a yellow-seeded oil-type yellow sarson and a black-seeded vegetable-type pak choi. We identified 26 QTL regions across all 10 linkage groups for traits related to seed weight, seed germination and seedling vigor under non-stress and salt stress conditions illustrating the polygenic nature of these traits. QTLs for multiple traits co-localized and we identified eight hotspots for quantitative trait loci (QTL) of seed weight, seed germination, and root and shoot lengths. A QTL hotspot for seed germination on A02 mapped at the *B. rapa Flowering Locus C* (*BrFLC2*). Another hotspot on A05 with salt stress specific QTLs co-located with the *B. rapa Fatty acid desaturase 2* (*BrFAD2*) locus. Epistatic interactions were observed between QTL hotspots for seed germination on A02 and A10 and with a salt tolerance QTL on A05. These results contribute to the understanding of the genetics of seed quality and seeding vigor in *B. rapa* and can offer tools for *Brassica* breeding.

## Introduction

*Brassica rapa* (A genome, 2*n* = 20) consists of several economically important morphotypes, such as leafy vegetables, oilseed types and turnips, with huge morphological and genetic diversity. In recent years, the allopolyploid species *B. napus* (A and B genomes, 2*n* = 38) has replaced *B. rapa* as the main oilseed crop within the *Brassica* species*. B. rapa* annual oil seed crops like yellow sarson and brown sarson are still grown in regions with short seasons, but *B. rapa* is also used as an important source of genetic variation in *B. napus* improvement, especially in China and Australia (Chen et al., 2007, 2010; Rygulla et al., 2007). Besides the oil content of seeds for especially oil crops, good quality seed and vigorous seedling growth are important traits for crop establishment and higher yield in any crop (Kazmi et al., 2012; Khan et al., 2012). The protrusion of the radicle from the seed is termed seed germination, while seedling vigor refers to the ability of a seed lot to establish seedlings after seed germination under a wide range of environmental conditions (Foolad et al., 2007; Finch-Savage et al., 2010). Seed germination and seedling vigor are very complex traits influenced by different factors, such as the size and composition of the seed, physiological state of the seed, environmental effects during seed production, harvesting, processing and storage, and conditions during germination and early growth. Many efforts have been made to improve seed germination and seedling vigor by optimizing the non-genetic factors; however, the paradigm has shifted to investigate also the genetic factors and to use these to improve crop performance. In several species studies were done to identify quantitative trait loci (QTLs) for seed germination and seedling vigor traits under non-stress and abiotic stress conditions, e.g., in tomato (Foolad et al., 2007; Kazmi et al., 2012; Khan et al., 2012), rice (Wang et al., 2011, 2012), soy bean (Csanádi et al., 2001), wheat (Bai et al., 2013), barley (Mano and Takeda, 1997), *Arabidopsis* (Galpaz and Reymond, 2010; DeRose-Wilson and Gaut, 2011; Bouteillé et al., 2012), *B. napus* (Hatzig et al., 2015) and *B. rapa* (Dechaine et al., 2014). These studies have reported that seed germination and seedling vigor traits are regulated by many genes and are strongly affected by environmental conditions (Bettey et al., 2000; Koornneef et al., 2002; Finch-Savage et al., 2010).

Environmental conditions will vary in the presence and level of abiotic and biotic stresses that the seeds and seedlings have to cope with. Therefore, studies of seeds and seedlings need to be carried out under more than only optimal conditions, if they are to be relevant for practical growing situations. Salinity is one of the major limiting abiotic stresses for high crop production, affecting about 20% of agricultural land and 50% of irrigated land (Mittler, 2006; Ren et al., 2010; Dang et al., 2013; Su et al., 2013). High levels of salt especially during seed germination and early plant growth, directly affects the crop establishment, in severe cases leading to complete crop failure or strongly reduced yields (Mano and Takeda, 1997; Ashraf and McNeilly, 2004; Su et al., 2013). Salinity stress reduces the plant's ability for water uptake, causing osmotic stress. At the same time, the accumulation of ions leads to the disturbance of ion homeostasis of plant cells (Wang et al., 2012). Since germinating seeds and establishing seedlings are also vulnerable to salinity stress (Ashraf and McNeilly, 2004), crop establishment and yield can be greatly affected. Salinity tolerance is related to genetic variation (DeRose-Wilson and Gaut, 2011) and thought to be a complex phenomenon controlled by many genes (Ouyang et al., 2007; Galpaz and Reymond, 2010; Joosen et al., 2010; DeRose-Wilson and Gaut, 2011). For a number of crops, it has been established that larger seed size and higher seed weight indicate more reserve food and contribute positively to seedling establishment (Ellis, 1992; Khan et al., 2012). For the *Brassica* genus, traits such as seed and seedling weight, and seed germination were primarily studied in *B. napus* rather than *B. rapa* (Hatzig et al., 2015). However, for *B. rapa*, knowledge about genomic regions responsible for seed germination and seedling vigor is largely lacking, except for a recent study where QTLs for traits related to reproductive fitness with a focus on percentage of germination, seed color, days to bolting and silique related traits (Dechaine et al., 2014). In this study, a *B. rapa* doubled haploid (DH) population from a cross of an oilseed yellow sarson and a vegetable pak choi was used to study the genetics of seed weight, seed germination and seedling vigor.

We identified 26 QTL regions for traits related to seed weight, seed germination and seedling vigor under non-stress and stress conditions and QTLs for multiple traits co-localized. We identified the candidate genes *B. rapa Flowering Locus C* (*BrFLC2*) and *B. rapa Fatty acid desaturase2* (*BraFAD2*), homologs of the *A. thaliana FLC* and *FAD2* genes, based on co-location of their expression QTLs (eQTLs) with germination and seedling vigor QTL hotspots and supported by their described functions in related species.

## Materials and methods

### Plant material and growing conditions

A *B. rapa* progeny of 170 DH lines (DH68) was developed from three F_1_ plants of a cross of a yellow sarson (YS143; accession number: FIL500) and a pak choi (PC175 cultivar: Nai Bai Cai; accession number: VO2B0226) (Xiao et al., 2013). Yellow sarson is a self-compatible annual oil crop with yellow seed color, while pak choi is a self-incompatible leafy vegetable with black seed color.

This DH68 population was sown in the greenhouse on a single day on 25th January 2010 (18°C/16°C day/night temperature, 80% humidity and 16 h day light). The DH lines varied in time to flowering (43–99 days after sowing) and thus seed maturation was non-synchronous. In 2011, the DH68 population was sown again, this time however, at five different dates from the second week of January to the last week of March. The aim of this staggered sowing was to have flowering of all the lines in the same period in order to avoid different environmental conditions during seed development. As a result all the DH lines started flowering during the first 2 weeks of April, 2011 (31–76 days after sowing). The harvested seeds were stored at 13°C temperature and 30% relative humidity. Germination and seedling vigor experiments were carried out with seeds of 120 DH lines for which enough seeds were available. In addition, 1000-seed weight, which reflects seed content and seed size, was measured by weighing 300–500 seeds of each genotype and converting to the weight of 1000 seeds.

### Pilot study to select optimal NaCl concentration for the salt stress experiments

A pilot study was conducted to determine the optimum level of NaCl concentration for the evaluation of salt stress. For the two parental lines and a small subset (5–7 DH lines) of the DH population, seed germination and root- and shoot- lengths were initially screened by germinating 30 seeds per genotype in petridishes with two layers of filter papers soaked in seven different NaCl concentrations: 10, 15, 25, 50, 75, 100, and 150 mM NaCl. In case of seedling vigor assay, root and shoot lengths were measured at 1, 3, 5, 7, and 9 days after germination (DAGs).

Seed germination and root and shoot length under salt stress conditions at 10, 15, and 25 mM NaCl were comparable to that under non-stress (0 mM NaCl), indicating that these concentrations were too low to induce visible symptoms of salt stress. At the concentration of 100 mM NaCl, seeds hardly germinated; at 75 mM NaCl there was germination, but the seedlings did not grow out enough to be able to measure root and shoot length. Therefore, in this study, we used 0 mM NaCl (non-stress) and 50 mM NaCl (salt stress) for phenotyping the DH population. As roots and shoots had hardly grown at one DAG and showed very little variation among DH lines, it was decided to measure these traits at 3, 5, 7, and 9 DAGs under both non-stress and salt stress. The materials and methods used for media preparation, seed germination and seedling growth are described in the following sections.

### Germination conditions and seed sterilization

Seeds harvested in 2010 and 2011 were used to assess seed germination of the DH lines. Seed germination experiments were conducted in petridishes on two layers of filter papers soaked with agar (non-stress; 0 mM NaCl) or 50 mM NaCl solution (salt stress). The solutions were autoclaved at 120°C and 1.5 bar for 18 min. Seeds were sterilized by keeping the seeds overnight in a closed container in chlorine gas fumes of a solution of 20 ml demi-water, 3 ml of 37% fuming HCl and 80 ml of 12% NaOCl. Per treatment per DH line, one set of 30 sterilized seeds was transferred to a petridish. Seeds were all placed between 16:00 and 18:00 h, so that radicle protrusion would start in the morning of the next day. The petridishes were placed in a climate room (21°C) with 16/8 h light/dark conditions in a completely randomized design. Seeds were considered to have germinated when the radicle protrusion had occurred. Starting the next day, the number of germinated seeds in each petridish was counted five times per day in 3-h intervals from 9:00 to 21:00 until all seeds had germinated.

### Seedling vigor assay

Seeds harvested in 2010 and 2011 were used to assess seedling vigor of the DH lines. Seedling vigor was measured by placing germinated seeds on vertical plates with 0.8% agar-medium without NaCl (non-stress) or with 50 mM NaCl (salt stress). About 80–90 ml of the agar medium was poured into rectangular plates (12 × 12 × 1.7 cm) in a laminar flow-cabinet. The top one-third portion of agar was removed to leave space for shoot growth. Germinated seeds from the DH lines and parental accessions were transferred from petridishes of the germination assay onto the agar edges of the vertical plates so that all the seedlings in a plate were in the same phase of germination. In total, 15 seeds per DH line (five seeds per plate, three replicate plates per DH line) were transferred and spaced equally. The plates were sealed with plastic foil and placed in a slanting position at a 60° angle to keep plants growing vertically and to avoid covering of the plates with transpired moisture. All the plates were placed in a climate chamber (21°C temperature, 16/8 h of light/dark) according to a randomized complete block design (replicates as blocks). Since the study focused on seed germination and early stages of seedling establishment, the seedlings were grown for only the first 10 DAGs. Seedling vigor was quantified by measuring the lengths of shoot and root at different DAG (during first 10 DAGs) and weighing fresh and dry weight of root and shoot per DH line at 10 DAG under both non-stress and salt stress conditions.

In the 2010 assay, the seedlings were grown for 10 days, and root length was measured at 3, 5, 7, and 9 DAGs while the shoot length was measured at 3 and 5 DAGs. The root and shoot lengths were measured manually at 3 and 5 DAGs, while image analysis was also done to measure root length at 3 and 5 DAGs for calibration against the manual measurements and then continued to 7 and 9 DAGs. For image analysis, photos were taken with a digital camera (Nikon D80) as described in Joosen et al. (2010). The root length from the digital image was analyzed using the EZ-Rhizo software package following the procedure described by Armengaud et al. (2009). In the assay of 2011, root and shoot length were measured only manually with a ruler at 3, 5, 7, and 9 DAGs.

### Seedling dry weight and fresh weight measurements

At 10 DAG, seedlings were taken out from the agarose-gel and rinsed with water to remove the agar from the roots. Root and shoot were separated and wrapped in white tissue paper for 2 h to absorb adhering water before determination of fresh weights. Root or shoot samples of DH lines were pooled over all seedlings of three replicates before taking the weight in order to avoid measurement error due to a too low weight of the samples. For each DH line, roots and shoots were dried overnight at 105°C, then dry weights were measured.

### Statistical data analyses

#### Calculation of seed germination parameters

A non-linear germination curve was fitted for each DH line using the Hill function (El-Kassaby et al., 2008) in the software package Germinator (Joosen et al., 2010); growth curves were not fitted for root and shoot length because of the limited number of time points (only 4 time points: 3, 5, 7, and 9 DAGs). Five germination parameters were estimated from the non-linear germination curves: the onset of germination (T10: time to reach 10% germination, in hr), the rate of germination (T50: time to reach 50% germination, hr), uniformity of germination (U7525: time between 25 and 75% germination, hr), maximum germination (Gmax: maximum germination, %) and area under the germination curve (AUC: area under curve) between time zero and 68 hr, the latest time point in this study; higher values for AUC correspond to earlier germination and a higher germination.

#### Calculation of salt tolerance parameters

In order to assess the performance of the DH lines for root or shoot length under non-stress and salt stress conditions, two different parameters were used: relative salt tolerance (RelST) and a salt tolerance index (STI) (Saad et al., 2014). RelST is the ratio of a trait value under salt stress vs. non-stress conditions (see the formula below), and indicates the relative performance of genotypes for their root, or shoot growth across conditions. A genotype with RelST greater than one for root length has a longer root under salt stress than under non-stress; a genotype with RelST lower than one is sensitive to salt as illustrated by reduced root length under salt stress. A value of one for RelST indicates that the root length of the genotype is not affected by the stress. STI was calculated by comparing the shoot or root length under stress and non-stress conditions, but now relative to the average length under the non-stress condition over the whole population using the formula below, as described by Fernandez (1992). An STI equal to one indicates that root or shoot length of a specific genotype under stress/non stress is equal to the average length under the non-stress condition over all DH lines. An STI greater than one indicates that the root/shoot length of a DH line is higher in one condition, or in both conditions relative to the mean of the population under non-stress conditions. If the STI is lower than one, there is lower root/shoot length in one or both conditions as compared to the average population under non-stress conditions.

$$\begin{array}{l} {\text{RelST}}_{\text{ij}}=\frac{{\text{X}}_{\text{ij}}\text{ at stress}}{{\text{X}}_{\text{ij}}\text{ at non-stress}}\\ \;{\text{STI}}_{\text{ij}}=\frac{\left({\text{X}}_{\text{ij}}\text{ at }{\text{non-stress}}^{*}{\text{X}}_{\text{ij}}\text{ at stress}\right)}{{\left({\text{X}}_{\text{average }(\text{j})}\text{ at non-stress}\right)}^{\text{2}}} \end{array}$$

where X_ij_ = root or shoot length of genotype i at j days after germination (DAG).

#### Summary statistics, graphical representation and heritability

Descriptive statistics were calculated for all traits. Box plots were made to visualize the distributions of seed germination parameters across the experiments, and shoot and root length across growing days and experiments.

Separate heatmaps were generated to visualize the Pearson correlation coefficients among seed germination parameters or shoot and root lengths at different DAGs for two treatments and using seed batches of two years. The heatmaps of the correlations were combined with hierarchical clustering of the traits using Euclidean distances and complete linkage after scaling the traits to zero mean and standard deviation one (equivalent to clustering on the Pearson correlations).

As DH lines are genetically fixed homozygotes, there is no variation due to dominance and therefore, narrow-sense heritability was estimated as: *h*^2^= $\sigma_{\text{a}}^{2}$/($\sigma_{\text{a}}^{2}$ + $\sigma_{\text{e}}^{2}$), where h^2^ is narrow-sense heritability, $\sigma_{\text{a}}^{2}$ is additive genetic variance and $\sigma_{\text{e}}^{2}=$ environmental variance (Bernardo, 2002). The variance components ($\sigma_{\text{DH}}^{2}$ and $\sigma_{\text{e}}^{2}$) were estimated using a linear model (One-way ANOVA): trait = DH line + replication + error. The additive genetic variance ($\sigma_{\text{a}}^{2}$) was estimated as $\sigma_{\text{a}}^{2}=\sigma_{\text{DH}}^{2}$/2 (Bernardo, 2002). The computation of Pearson correlation coefficients, the hierarchical clustering, heritability calculation and heatmap visualization were performed using R statistical software (R Core Team, 2012).

### Genetic map construction

Linkage analysis and map construction were performed with JoinMap 4.0 (Van Ooijen, 2006) using a regression approach and the Kosambi map function. In total 435 markers mainly AFLPs, SSRs, and *Myb* targeted markers and gene-targeted markers were mapped in an integrated map of 10 linkage groups. This integrated map is a slightly modified version of the linkage map presented in Xiao et al. (2013), as additional DH lines from two different F_1_ plants of the same cross were used in the present paper. As the parents were not homozygous, these F_1_ plants were not identical, which resulted in minor changes in the genetic maps (Supplementary Table S2).

### QTL analysis

Single trait QTL analysis was performed to identify genomic regions controlling a trait, using interval mapping (IM), and restricted and full multiple QTL model mapping (rMQM and MQM) in MapQTL 6.0 (Van Ooijen, 2009). Initially peak markers from a map region with LOD score >2 were used as cofactors and a final list of cofactors was selected using the automatic cofactor selection procedure, which uses a backward elimination approach. The cofactor selection process was repeated with different sets of cofactors until the QTL profile was stable.

QTL mapping was carried out for all the germination parameters, root and shoot length, and fresh and dry weight of both the root and the shoot. Similarly, QTL mapping was done for the salt tolerance index and for relative salt tolerance of root and shoot length at different DAGs. In this study, a genome-wide significance LOD score threshold of 3.0 was derived at the 95 percentile of 10,000 permutations of each trait. There was hardly any variation in the threshold between traits so, this threshold was used for all traits to declare a QTL as significant. QTLs with a LOD score between 2 and 3 were considered as putative QTLs. Finally, 1-LOD support intervals were determined for the assigned QTLs. The cut-off value for declaring a number of co-locating QTLs as a hotspot was calculated using the package “hotspots” in R (Darrouzet-Nardi, 2010). In this study, co-localized QTLs were coded as “*Co-QTLk-m*,” where *k* indicates for linkage group and *m* for QTL number.

Based on the observed main effects of significant or putative QTLs, epistatic interactions were tested for all possible pairs of two QTLs using the following ANOVA model: trait_i_ = QTL_1_ + QTL_2_ + ${\text{QTL}}_{1}^{*}$QTL_2_+ error. First, an ANOVA model consisting of only the main effects of the QTLs was fitted. Then, the QTL^*^QTL interaction term was added, and this change to the model was tested for significance; if significant, the contribution of this epistatic interaction to the phenotypic variance was quantified. ANOVA were performed in R.

### Quantitative real-time PCR (RT-qPCR) and eQTL analysis

Transcript abundance of candidate genes *BrFLC2* and *BrFAD2*, was determined in developing seeds of the DH population using RT-qPCR. Transcripts of the genes were profiled with two technical replicates using RNA samples of seeds harvested 28 days after pollination of the 120 DH lines. RNA isolation and purification were done following the same protocol used by Basnet et al. (2013). Transcript abundance of these genes was measured in RT-qPCR in 96-well optical reaction plates using the iQ™ SYBR® Green Supermix (Bio-Rad, www.Bio-rad.com) according to Xiao et al. (2013), but actin was used as reference gene to calculate the cycle threshold (*Ct*) values and ΔΔ*Ct* values. eQTL analyses were done using interval mapping (IM), and restricted and full multiple QTL model mapping (rMQM and MQM) in MapQTL 6.0 (Van Ooijen, 2009). Molecular markers specific for *BrFLC2* and *BrFAD2* genes were mapped in this population; an eQTL was defined as a *cis*-eQTL (local eQTL) if the edge of a 2-LOD support interval of an eQTL was within 10 cM of the genetic map position of the gene, otherwise the eQTL was defined as a *trans*-QTL (distant eQTL).

## Results

### Seed weight and seed germination

Yellow sarson had larger and heavier seeds, which germinated earlier (lower T10) and at a faster rate (lower T50) than pak choi seeds; generally, germination under non-stress was earlier and more uniform than under salt stress (Table 1). Thousand-seed weight was almost three times higher for yellow sarson than for pak choi. The Gmax of two parental genotypes and DH lines varied from 26.7 to 100% across conditions (Table 1).

**Table 1:** **Summary statistics of seed germination parameters under non-stress and salt stress conditions of 2010 and 2011 seed batches**.

**Traits**	**Treatment**	**2010**				**2011**			
		**Parents**		**DH lines**		**Parents**		**DHlines**	
		**PC175**	**YS143**	**Mean ± SD**	**Range**	**PC175**	**YS143**	**Mean ± SD**	**Range**
Gmax (%)	Control	100.0	100.0	97.6±10.3	26.7–100	96.5	100.0	96.4±7.7	45.7-100
	50 mM	100.0	100.0	96.5±9.0	50.0–100	100.0	100.0	95.4±10.4	32.3-100
U7525 (h)	Control	1.5	1.0	4.2±3.6	0.4–22.7	2.5	2.2	7.8±5.1	1.4-30.9
	50 mM	1.3	0.7	4.4±3.2	0.7–16.8	4.0	7.9	9.2±5.7	1.8-33.2
T50 (h)	Control	19.0	15.3	21.9±5.1	11.1–44.7	19.3	15.1	21.4±6.9	10.0-48.6
	50 mM	17.8	17.0	24.4±6.3	15.1–52.5	22.1	13.9	26.1±10.4	11.4-79.2
T10 (h)	Control	17.6	14.3	18.5±4.3	7.0–40.5	16.9	13.0	14.8±4.3	4.7-26.3
	50 mM	16.5	16.3	20.5±5.4	11.3–41.2	18.5	8.0	17.9±6.0	7.8-42.3
AUC	Control	81.0	84.7	75.9±10.1	13.7–88.1	75.6	84.8	73.5±11.0	26.2-88.6
	50 mM	82.2	83.1	72.7±10.9	29.9–84.9	76.5	84.5	68.9±13.0	15.5-88.0
Seed weight^*^	–	1.9	6.6	2.5±0.9	0.5–5.9	1.4	5.8	2.2±0.8	0.2 - 5.3

* *1000 seed weight in gram (g)*.

In the DH population, seed germination started earlier (lower average T10) and was more uniform (lower U7525) under non-stress than salt stress. Germination rate (T50) and uniformity (U7525) were positively correlated to each other, and negatively with Gmax and AUC (Supplementary Figure S1). Pearson correlation coefficients of the same parameter were higher between two seed batches (two growing years) than between stress levels (Supplementary Figure S1). Thousand-seed weight was positively correlated with AUC (*r* = 0.25 to 0.37) and Gmax (*r* = 0.16 to 0.24) and negatively correlated with T10 (*r* = −0.18 to −0.36), T50 (*r* = −0.25 to −0.31) and U7525 (*r* = −0.11 to −0.24) under non-stress and salt stress conditions across 2 years' seed batches, indicating faster germination and higher germination percentages of heavier seeds.

### Root and shoot length of seedlings

**Figure 1:**
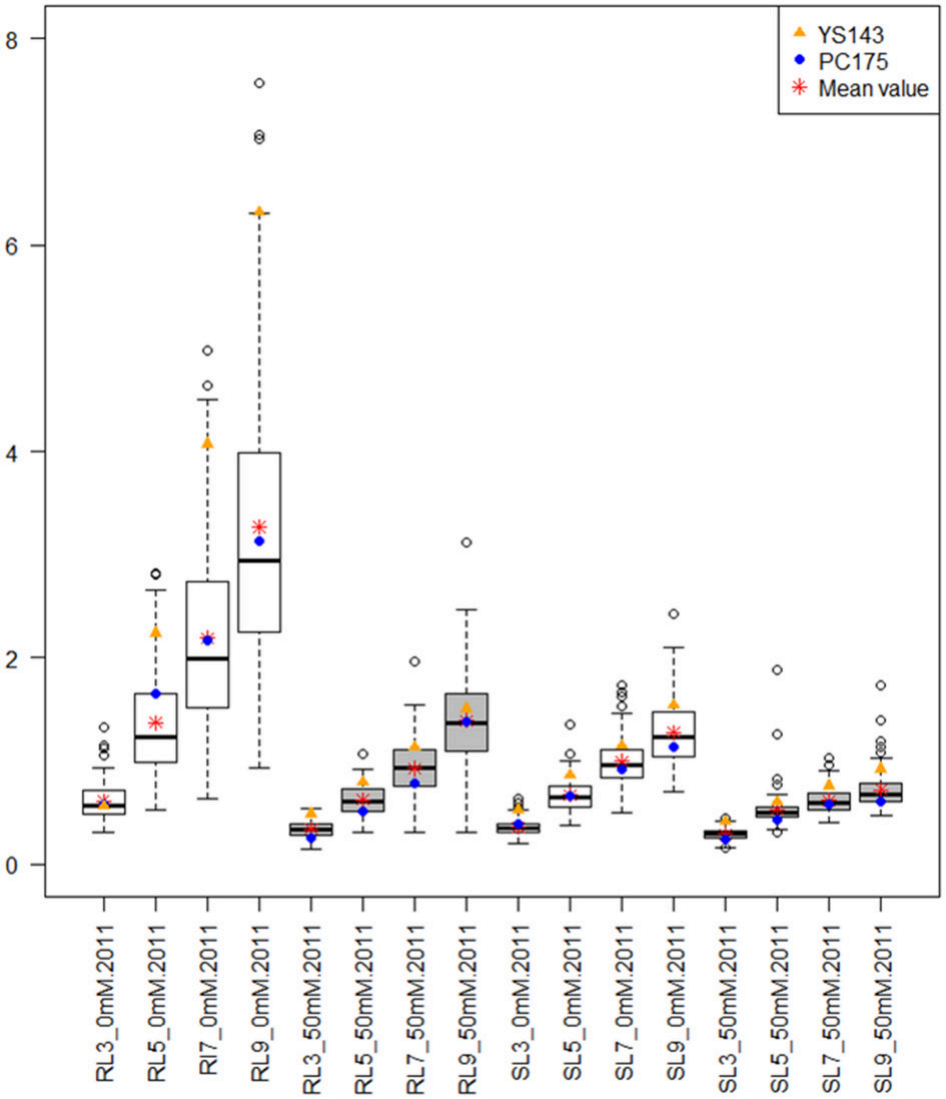
**Box plots showing the distributions of root length (RL) and shoot length (SL) at different days after germination (DAG) under non-stress (0 mM NaCl) and salt stress (50 mM NaCl) for the 2011 seed batch**. The shaded color of the boxes indicates the treatments: white for non-stress and gray for salt stress. The y-axis indicates root and shoot length (in cm). The x-axis label is the combination of RL or SL at 3, 5, 7, and 9 DAG at non-stress and salt stress conditions. Box plots showing the distributions of RL and SL for the 2010 seed batch are shown in Supplementary Figure S2.

The roots of yellow sarson were longer than roots of pak choi (*p* = 0.05) on all DAGs and the differences in root length between the parents increased over time. The variance of root- and shoot- length over the DH lines also increased with time (Figure 1; Supplementary Figure S2). Under salt stress root length was reduced (Figure 1; Supplementary Figure S2); yellow sarson had longer roots than pak choi until 7 DAGs, however at 9 DAG root lengths of the parental genotypes were similar. Similar to root length, also shoot length was larger in yellow sarson than pak choi and the difference between the two parents was smaller under salt stress than under non-stress conditions. Large variation in shoot length was observed across the DH lines. In both conditions in both years, a large number of transgressive segregants were observed across the DH population for all the seedling traits (Figure 1; Supplementary Figure S2).

Both fresh and dry weight of root and shoot were higher in yellow sarson than in pak choi, except for root fresh and dry weight under salt stress of the 2011 seed batch (Table 2). Fresh and dry weight of root and shoot decreased under salt stress for the parents as well as the DH population, and again the decrease was stronger for yellow sarson than for pak choi.

**Table 2:** **Summary statistics of root and shoot length, and their fresh and dry weight under non-stress and salt stress conditions of 2010 and 2011 seed batches**.

**Trait**	**Year**	**Day**	**Treatment**	**Root**				**Shoot**			
				**Parents**		**DH lines**		**Parents**		**DH lines**	
				**PC175**	**YS143**	**Mean ± SD**	**Range**	**PC175**	**YS143**	**Mean ± SD**	**Range**
Length (cm)	2010	3	Control	0.6 ± 0.1	1.6 ± 0.1	1.3 ± 0.6	0.2–3.5	0.5 ± 0.1	0.6 ± 0.1	0.6 ± 0.2	0.3–1.5
		3	50 mM	0.5 ± 0.1	0.7 ± 0.1	0.5 ± 0.2	0.1-1.2	0.4 ± 0.1	0.5 ± 0.1	0.4 ± 0.1	0.2–0.9
		5	Control	1.3 ± 0.9	5.6 ± 0.1	2.3 ± 1.0	0.3–6.7	0.9 ± 0.2	1.5 ± 0.2	1.0 ± 0.4	0.5–2.7
		5	50 mM	1.1 ± 0.2	1.3 ± 0.4	0.9 ± 0.3	0.2–1.8	0.6 ± 0.1	0.7 ± 0.1	0.5 ± 0.1	0.2–1.4
		7	Control	1.9 ± 0.3	7.0 ± 0.1	2.8 ± 1.2	0.7–7.3	–	–	–	–
		7	50 mM	1.8 ± 0.2	1.8 ± 0.7	1.2 ± 0.4	0.3–2.6	–	–	–	–
		9	Control	1.9 ± 0.3	7.2 ± 0.2	3.3 ± 1.4	0.8–8.6	–	–	–	–
		9	50 mM	2.4 ± 0.2	2.5 ± 1.6	1.5 ± 0.6	0.3-3.9	–	–	–	–
	2011	3	Control	0.6	0.6	0.6 ± 0.3	0.1–2.1	0.4	0.5	0.4 ± 0.1	0.1–0.9
		3	50 mM	0.3	0.5	0.3 ± 0.1	0.1–0.7	0.2	0.4	0.3 ± 0.1	0.1–0.5
		5	Control	1.7	2.2	1.4 ± 0.8	0.1–6.2	0.7	0.9	0.7 ± 0.2	0.1–2.0
		5	50 mM	0.5	0.8	0.6 ± 0.2	0.1–2.0	0.4	0.6	0.5 ± 0.1	0.1–1.0
		7	Control	2.2	4.1	2.2 ± 1.3	0.2–9.0	0.9	1.2	1.0 ± 0.4	0.1–3.0
		7	50 mM	0.8	1.1	0.9 ± 0.4	0.2–3.5	0.6	0.8	0.6 ± 0.2	0.1–1.7
		9	Control	3.1	6.3	3.3 ± 1.9	0.3–12.0	1.1	1.5	1.3 ± 0.5	0.4–3.0
		9	50 mM	1.4	1.5	1.4 ± 0.7	0.2–0.4	0.6	0.9	0.7 ± 0.2	0.3–2.5
Weight (g)	2010	Fresh	Control	70.0	110.0	69.8 ± 46.2	11.4–276.6	357.3	770.0	440.7 ± 133.7	158.6–813.3
			50 mM	25.2	40.6	13.5 ± 11.0	1.5–72.0	311.2	327.6	226.5 ± 88.8	57.0–503.0
		Dry	Control	9.8	21.0	16.0 ± 15.3	2.1—124.8	29.2	72.2	43.0 ± 19.6	15.5–114.8
			50 mM	5.8	7.2	4.2 ± 1.9	0.5—10.3	24.6	45.7	25.7 ± 10.7	3.0–51.5
	2011	Fresh	Control	90.0	130.0	65.5 ± 33.2	13.9—199.3	400.0	540.0	428.0 ± 98.5	151.1–687.5
			50 mM	17.1	7.2	29.1 ± 21.9	2.5—141.4	246.0	353.6	325.7 ± 89.7	90.0–560.5
		Dry	Control	7.7	17.4	8.2 ± 0.3	2.9—17.4	26.7	80.9	34.4 ± 12.2	5.3–86.6
			50 mM	5.6	4.3	4.5 ± 0.2	0.1—11.7	19.0	60.7	26.9 ± 9.2	11.4–60.7

*”–“ Not available*.

### Cluster analysis of root and shoot traits

In a hierarchical cluster analysis among the traits using Pearson correlation coefficients, two main clusters were observed, one for root traits, the other for shoot traits, with generally low correlation coefficients between the two clusters (Supplementary Figure S3). Within both the root and shoot trait clusters, sub-clusters with stronger correlations were observed for the treatments followed by years. So, in the cluster analysis first the tissues are separated (root vs. shoot), then treatments, then seed batches/years.

### Heritability

Root and shoot length under non-stress and stress conditions and the salt tolerance parameters STI and RelST had low to moderately high narrow-sense heritabilities (0.2–0.7) (Table 3). Under salt stress, heritabilities were generally lower than under control conditions. The heritabilities were generally higher for the seed batch of 2011, where flowering and seed ripening were synchronized, than for the seed batch of 2010.

**Table 3:** **Narrow-sense heritabilities of root length (RL), shoot length (SL) and salt tolerance parameters under non-stress and salt stress conditions of 2010 and 2011 seed batches for different days after germination (DAGs)**.

	**DAG**	**Control**		**50 mM NaCl**		**2010**		**2011**	
		**2010**	**2011**	**2010**	**2011**	**RelST**	**STI**	**RelST**	**STI**
Root length	**3**	0.6	0.6	0.5	0.6	0.3	0.5	0.2	0.6
	**5**	0.6	0.6	0.6	0.6	0.5	0.5	0.2	0.6
	**7**	0.6	0.7	0.6	0.6	0.5	0.5	0.2	0.6
	**9**	0.6	0.7	0.53	0.6	0.4	0.4	0.4	0.6
Shoot length	**3**	0.6	0.6	0.4	0.5	0.3	0.6	0.2	0.6
	**5**	0.5	0.6	0.4	0.5	0.3	0.5	0.3	0.6
	**7**	–	0.7	–	0.6	–	–	0.5	0.6
	**9**	–	0.7	–	0.7	–	–	0.5	0.7

*RelST indicates Relative Salt Tolerance, STI indicates the Salt Tolerance Index*.

### QTLs for seed germination and seed weight

**Figure 2:**
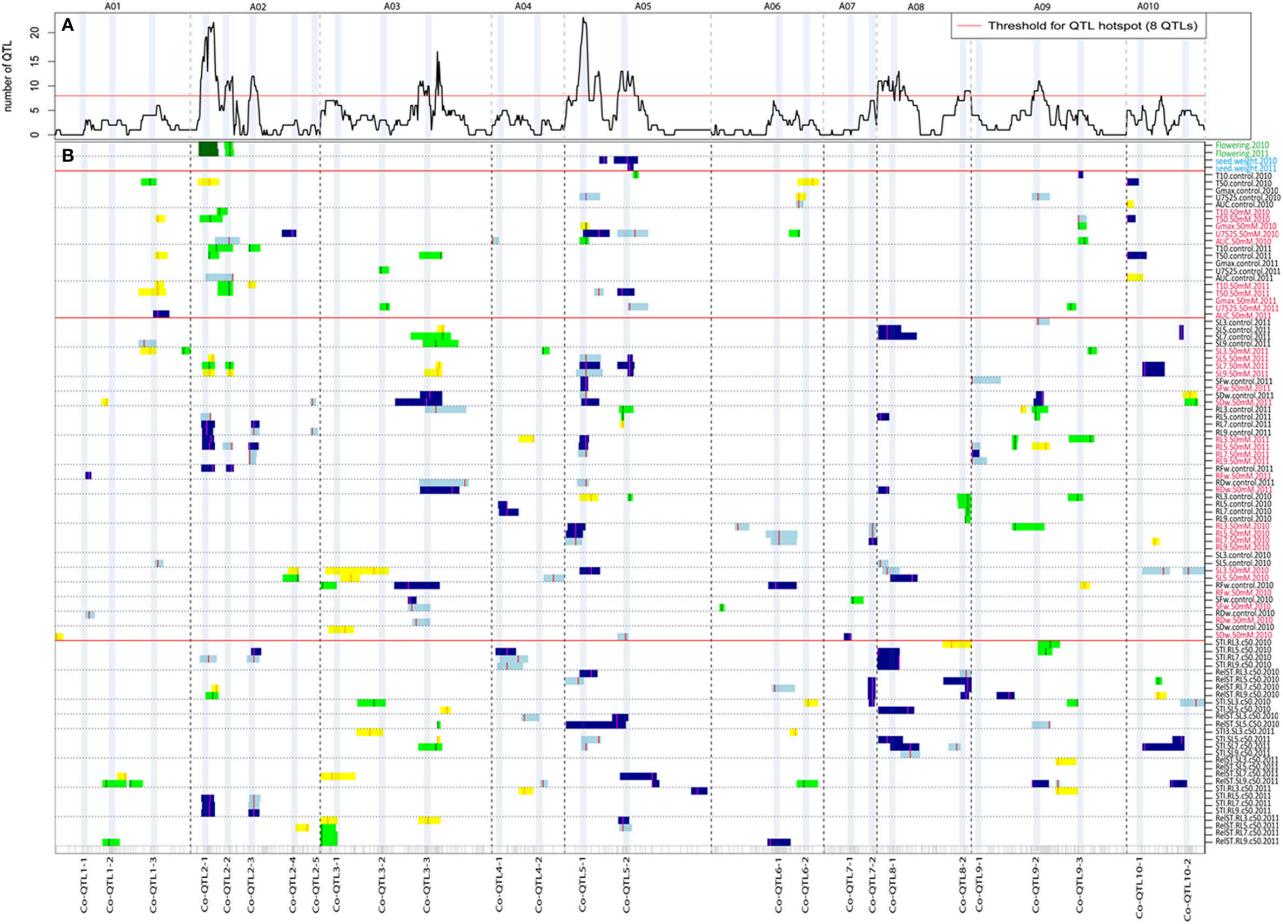
**An overview of single trait QTL profiles of seed germination, seedling vigor and salt tolerance parameters under non-stress and 50 mM NaCl salt of 2010 and 2011 seed batches**. **(A)** QTL hotspots (>8 significant QTLs) indicated by the number of significant QTLs plotted for 10 linkage groups (A01-A10). The dotted vertical lines separate the 10 linkage groups. **(B)** Single trait QTL profiles of flowering time, seed weight, seed germination, seedling vigor, and salt tolerance parameters. Seed germination includes five parameters: T10, T50, U7525, Gmax and AUC; seedling vigor includes root and shoot length at different DAGs, and root and shoot biomass measured under non-stress and 50 mM NaCl salt stress conditions. The different traits are on different rows along the y-axis, the 10 linkage groups on the x-axis, separated by dotted lines. QTLs that had a high phenotypic value for the YS143 allele are in light blue (for QTLs with LOD 2–3) and blue (for QTL with LOD >3) while QTLs with a high phenotypic value for the PC175 allele are indicated in yellow (for QTLs with LOD 2–3) and green (for QTLs with LOD > 3). The color mark on the QTL profile indicates the QTL peak position. The 26 QTL co-localization regions are indicated as Co-QTL followed by the number of the linkage group and a serial number within a linkage group, for example, Co-QTL1-2 indicates the second co-localization QTL region on A01. The red color of a trait label indicates the trait under 50 mM NaCl salt stress. The trait labels are described in Supplementary Table S1. LOD scores of QTLs shown in this figure are also presented in Supplementary Table S6.

Over the two seed batches (2010 and 2011), two conditions (non-stress and salt stress) and five germination parameters, in total, 26 QTLs and 20 putative QTLs (LOD score between 2 and 3) were found (Figure 2B; Supplementary Table S3). For the significant QTLs, the explained variances ranged from 7.5 to 27.2%. On A02, QTLs were detected mainly for T10 and T50 (Supplementary Table S3), with the favorable effect coming from the yellow sarson allele. On A05, one QTL and three putative QTLs with explained variance from 6.5 to 11.1% were mapped for mainly uniformity under salt stress. On A10, 3 QTLs with explained variance from 10.3 to 15.4% were mapped mainly for T50 under both non-stress and salt stress conditions. For 1000-seed weight in 2010 and 2011 a single QTL was found on A05, with explained variance ranging from 8.3 to 16.1%.

### QTLs for seedling vigor

For the 24 seedling vigor traits measured in 2010 and 2011 seed batches, 10 QTL regions with 69 QTLs (for traits = combinations of traits/observation day/seed batch/salt treatment) were identified, distributed over 10 linkage groups (Figure 2B; Table 4; Supplementary Table S4). The explained variances ranged from 7.1 to 24.3%. For the 2010 seed batch, 22 QTLs for 13 traits were identified on different linkage groups with at least one QTL per trait and an additional 24 putative QTLs for 13 different traits (Figure 2B; Supplementary Table S4). Since root and shoot length traits in 2010 and 2011 were measured repeatedly at 3, 5, 7, and 9 DAGs, many QTLs for the same trait at different DAGs most likely represent the same QTL.

**Table 4:** **List of consensus QTLs for the traits related to flowering time, seed weight, seed germination and seedling vigor in *Brassica rapa***.

**Traits**	**Year**			**A01**			**A02**				**A03**			**A04**		**A05**		**A06**		**A07**		**A08**		**A09**			**A10**	
			**Trait symbol**	**Co-QTL1-1**	**Co-QTL1-2**	**Co-QTL1-3**	**Co-QTL2-1**	**Co-QTL2-2**	**Co-QTL2-3**	**Co-QTL2-4**	**Co-QTL3-1**	**Co-QTL3-2**	**Co-QTL3-3**	**Co-QTL4-1**	**Co-QTL4-2**	**Co-QTL5-1**	**Co-QTL5-2**	**Co-QTL6-1**	**Co-QTL6-2**	**Co-QTL7-1**	**Co-QTL7-2**	**Co-QTL8-1**	**Co-QTL8-2**	**Co-QTL9-1**	**Co-QTL9-2**	**Co-QTL9-3**	**Co-QTL10-1**	**Co-QTL10-2**
1000-seed	2010															1q	1q											
weight	2011																1q											
Flowering	2010						1q	1q																				
time	2011						1q	1q																				
**GERMINATION**																												
T10	2010		T10					1q									1q									1q		
	2011		T10					1q;1q	1q																			
T50	2010		T50			1q	1q																				1q;1q	
	2011		T50				1q	1q					1q				1q										1q	
U7525	2010		U7525							1q						1q			1q									
	2011		U7525									1q;1q														1q		
Gmax	2010		Gmax																							1q		
AUC	2010		AUC											1q		1q										1q		
	2011		AUC			1q																						
**SEEDLING VIGOR**																												
**Root**																												
Root	2010		RL											2q		1q;3q	1q	2q			3q		4q		1q	1q		
length	2011		RL				2q;2q	1q	1q;1q							2q	2q					1q		1q	2q	1q		
Root fresh	2010		RFw								1q		1q					1q										
weight	2011		RFw	1q			1q	1q																				
Root dry	2010		RDw																									
weight	2011		RDw										1q									1q						
**Shoot**																												
Shoot	2010		SL							1q						1q						1q						
length	2011		SL				1q	1q					2q		1q	1q	3q					2q				1q	2q	2q
Shoot	2010		SFw										1q							1q								
fresh weight	2011		SFw													1q;1q												
Shoot	2010		SDw																	1q								
dry weight	2011		SDw										1q;1q			1q									1q;1q			1q
**Salt tolerance**																												
Root	2010	STI	RL						1q					1q								3q			2q			
length	2011	STI	RL				3q		1q																			
	2010	RelST	RL				1q									1q					3q		3q					
	2011	RelST	RL		1q						3q						1q	1q										
Shoot	2010	STI	SL									1q									1q	1q				1q		
length	2011	STI	SL										1q									2q					1q	2q
	2010	RelST	SL										1q			1q	1q											
	2011	RelST	SL		1q												1q		1q						1q			1q

*“q” indicates a QTL for the traits, black font color indicates a QTL under non-stress (control) and red color for a QTL under salt stress condition. The number in front of “q” indicates the number of QTLs detected for RL/SL measured at different days after germination (DAG)*.

For the 2011 seed batch, 47 QTLs were identified for 20 traits with at least one QTL per trait and all those traits also had putative QTLs. For two traits, many QTLs were detected: for root length at 5 DAG under 50 mM salt in the 2011 seed batch, four QTLs with explained variance ranging from 8.3 to 13.9%, and for shoot length at 7 DAG under 50 mM salt for the 2011 seed batch, five QTLs with explained variance ranging from 10.0 to 14.2% (Figure 2B; Supplementary Table S4).

### QTLs for seedling salt tolerance parameters

The parameters STI and RelST were calculated for root and shoot lengths. In total, 47 QTLs (mainly co-localized on 8 regions (>5 QTLs)) were identified for 24 traits (root and shoot length at different DAGs in 2 years seed batches) with explained variance ranging from 7.8 to 22.2% (Figure 2B; Table 4; Supplementary Table S5). The trait RelST for shoot length at 9 DAG in 2011 had the largest number of QTLs (six) with explained variance from 11.7 to 17.7%. Since root and shoot length traits were measured repeatedly at 3, 5, 7, and 9 DAGs, many QTLs for the same trait at different DAGs probably represent the same QTL.

### Co-localization of QTLs

QTLs co-localized on 26 unique genomic regions across the 10 linkage groups; however eight QTL hotspots (with ≥ 8 QTLs) were detected (Figure 2A; Table 4; Supplementary Table S6). QTLs co-localized on these hotspots are often for the same traits measured at different DAGs, at different treatments or in different seed batches (years) (Figures 2A,B; Table 4; Supplementary Tables S3–S5). We also considered which parental origin of a QTL allele, yellow sarson or pak choi, could be of importance to breeders. QTL hotspots, such as *Co-QTL1-3* on A01, *Co-QTL2-1* and *-2* on A02 and *Co-QTL10-1* on A10 mainly included QTLs for T10 and T50 seed germination parameters, with the yellow sarson allele on A01 and A02 and the pak choi allele on A10 associated with earlier onset and faster germination (Figure 2B). On hotspots *Co-QTL2-1* and *-2* on A02, QTLs for root and shoot traits from the 2011 seed batch were also co-localized; here, the yellow sarson allele is associated with an increase in root length and the pak choi allele with increased shoot length. Hotspots *Co-QTL9-2* and *-3* contain QTLs associated with germination rate (T50), and Gmax and AUC under salt stress in 2010 seeds and uniformity (U7525) under salt stress in 2011 seeds; these hotspots also harbor a major QTL for seed color with 32.7% explained variation (Basnet et al., 2015). Besides QTL for germination under salt stress, these hotspots *Co-QTL9-2* and *-3* contain QTLs for shoot and root length and shoot weight under both conditions and also for salt tolerance parameters. At this locus, the pak choi allele was the favorable allele. On *Co-QTL8-1*, QTLs for root and shoot lengths and weight and salt tolerance parameter STI co-localize. Here, the yellow sarson allele was the favorable allele (Figure 2B).

### Stress treatment specific QTLs

The two QTL hotspots on A05 *Co-QTL5-1* and *-2* harbor QTLs for AUC and Gmax (in 2010), T50 (in 2011) and U7525 (in 2010 and 2011) under salt stress and RelST of root and shoot length in both years (Figure 2B). The pak choi allele increased the germination parameters Gmax and AUC from the 2010 seed batch, but is associated with a lower germination rate (T50 in 2011) and with a decreased uniformity of germination (U7525 in 2010 and 2011). The yellow sarson allele gave higher RelST and STI of root and shoot lengths (Figure 2B).

### Epistatic interactions between QTLs

**Figure 3:**
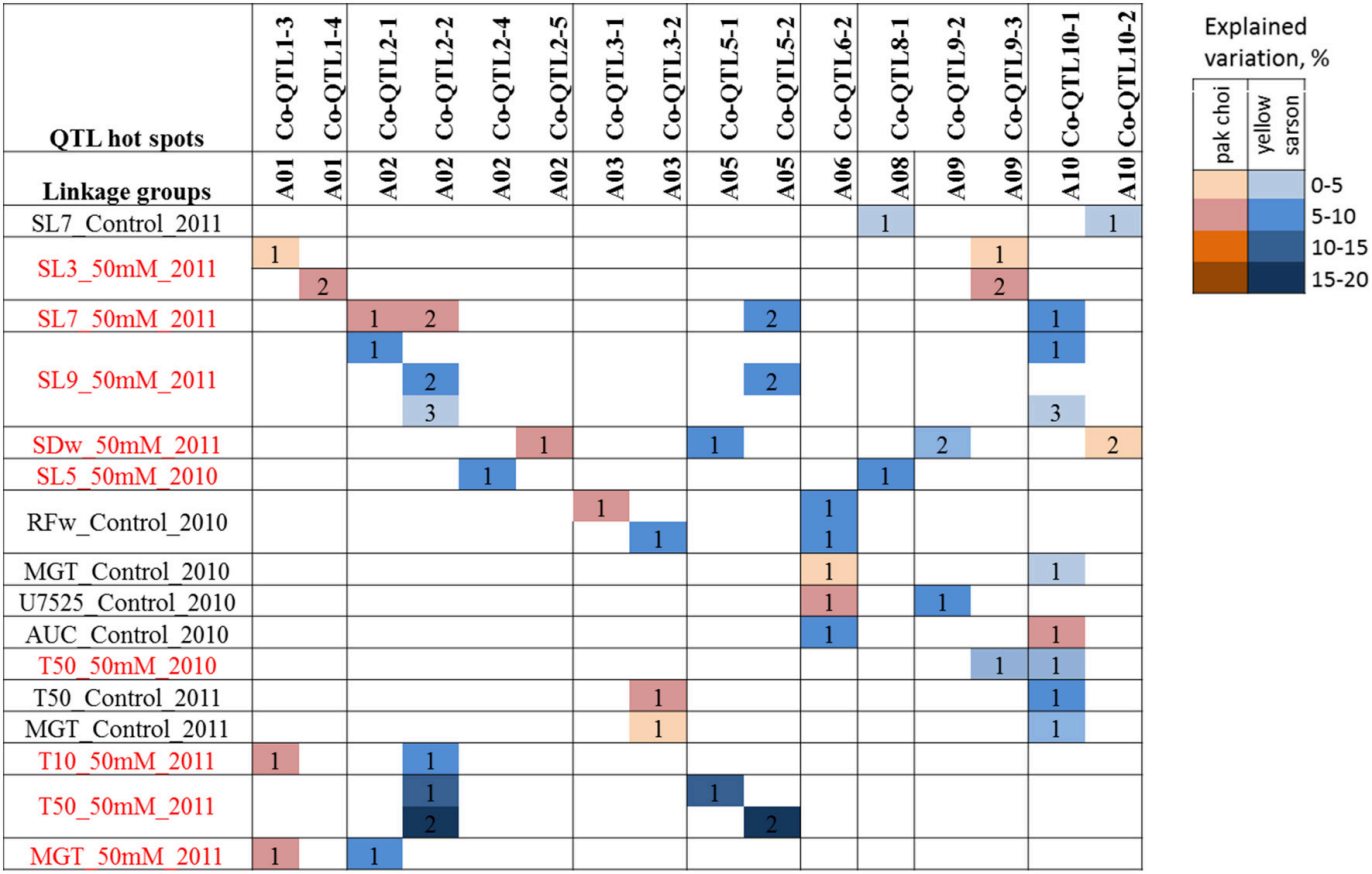
**Epistatic interactions of QTL regions for seed germination and seedling vigor traits**. QTLs identified for different traits were tested in a pair of two QTLs for each trait. Identical numbers within a trait-row indicate a pair of QTLs with significant epistatic interaction (α = 0.05) for that particular trait. The color intensity increases with higher explained variance (%) of the interaction.

Among the 64 trait combinations (germination and seedling vigor measured under two treatments from seeds harvested in 2 years), epistatic interactions were observed for 16 traits: 9 for germination and 7 for seedling vigor traits. Most had only a single epistatic interaction, with an explained variance between 5 and 10%; however, four traits had 2 or 3 epistatic interactions (Figure 3). For T50 (50 mM salt, 2011) there were two epistatic interactions between *Co-QTL2-2* and *Co-QTL5-1*, and between *Co-QTL2-2* and *Co-QTL5-2* with 10.54 and 17.58% explained variation, respectively. QTL hotspots *Co-QTL10-1, Co-QTL2-2* and *Co-QTL6-2* were the regions that had the largest number of QTL by QTL interactions, with 8, 6, and 5 other regions, respectively (Figure 3).

### Co-localization of phenotypic QTLs with candidate genes BrFLC2 and BrFAD2

*Co-QTL2-1* and *-2* on A02 co-localized with *BrFLC2, Co-QTL5-1* and *-2* co-localized with *BrFAD2* on A05. We analyzed transcript abundance of these two genes in the DH population in order to be able to map their expression QTLs (eQTLs).

**Figure 4:**
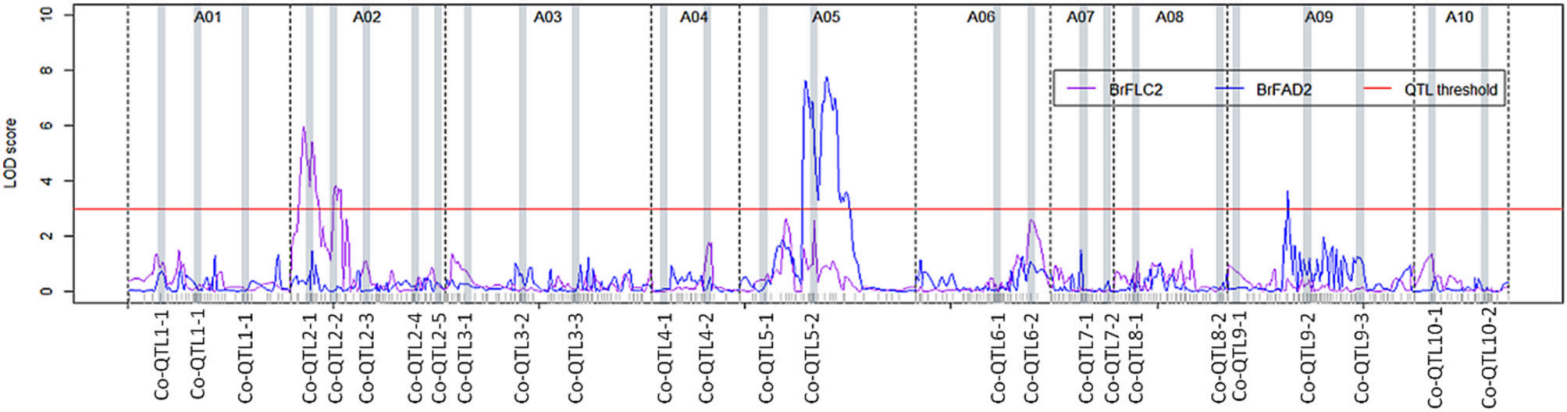
**eQTL profiles for ***BrFLC2*** (a ***Brassica*** homolog of ***FLC2*** of ***A. thaliana***) and ***BrFAD2*** (a ***Brassica*** homolog of ***FAD2*** of ***A. thaliana***) measured in developing ***B. rapa*** seeds (28 days after pollination) across 10 linkage groups**. The y-axis represents the LOD score, the x-axis represents the 10 linkage groups separated by dotted lines; the QTL significance threshold is indicated by a red colored solid line at LOD score 3.

For *BrFLC2*, a *cis*-eQTL was mapped over the *Co-QTL2-1* and *-2* hotspots on A02 with LOD scores 5.4 and 3.7 that explained 20.8 and 14.0% of the total variation in transcript abundance, respectively. For *BrFAD2*, a *cis*-eQTL co-located with the *Co-QTL5-1* and *-2* regions with LOD score 7.1 and 22.0% explained variance while another *trans*-eQTL mapped at the *Co-QTL9-2* region on A09 with LOD score 3.6 and 10.5% explained variance (Figure 4; Table 5).

**Table 5:** **Summary of expression QTLs (eQTLs) of ***BrFLC2*** and ***BrFAD2*** genes identified using interval mapping (IM) and multiple QTL mapping (MQM) in this DH population**.

**Gene name**	***A. thaliana* orthologs**	**Peak marker**	**Linkage group**	**Peak marker**		**2-LOD support interval**		**% Explained variation**	**Total variation explained**
				**Position (cM)**	**LOD**	**Lower position**	**Upper position**		
*BrFLC2*	*FLC2*	BRH04D11flc2	2	17.4	5.4	6.5	20.8	19.8	33.8
		BrPIP1b	2	37.9	3.7	33.1	42.7	14.0	
*BrFAD2*	*FAD2*	Myb2HaeIIIM-605.3	5	78.2	7.1	56.1	89.1	22.0	32.5
		BrFRY1P1b	9	51.5	3.6	48.1	57.6	10.5	

## Discussion

Good seed germination and high seedling vigor under diverse conditions are essential for the establishment of a crop. In this study, seed germination and early seedling vigor were evaluated in *B. rapa*: 120 genotypes from a DH population from a cross of *B. rapa* yellow sarson and brown seeded pak choi were used to identify genomic regions associated with seed germination characteristics and seedling vigor under non-stress and salt stress conditions. Seed germination and shoot and root related traits during the first 10 days after germination were assessed to quantify seed- and seedling- vigor. Twenty-six QTL regions for traits related to seed weight, seed germination and seedling vigor under non-stress and stress conditions were detected, which illustrates the polygenic nature of germination and seedling vigor traits. We identified eight major hotspots where QTLs for germination, seedling vigor and/or salt tolerance parameters co-located. Epistatic interactions were detected among several of the QTL hotspots.

### Early seedling growth is more affected by salt stress than seed germination

Under natural conditions, plants are exposed to different levels of salt stress; in this study 50 mM NaCl was chosen to mimic the salinity stress that affects seed germination and seedling vigor in a field situation. Maximum seed germination was not drastically affected at 50 mM NaCl, being still at a level of 95–100%; however, in general, other germination parameters, relating to rate and uniformity of germination were negatively affected. Root and shoot growth were also reduced.

### Oil-type yellow sarson has improved seed germination and seedling vigor compared to vegetable-type pak choi under non-stress and salt stress conditions but is more sensitive to salt stress

The yellow sarson parent had larger seed size and higher 1000-seed weight than the pak choi parent, and displayed earlier onset, more uniform and faster germination under both non-stress and salt stress conditions (Table 1). This parent also had a higher root- and shoot- length and biomass (Table 2). The positive correlations of 1000-seed weight with the germination parameters assessed in the DH population supports that larger seeds germinate earlier, faster, more uniformly and to a higher maximum germination than smaller seeds. Thus, we conclude that yellow sarson had higher seed quality and seedling vigor than pak choi. The explanation for the larger seeds of yellow sarson (than those of pak choi) could be that yellow sarson was selected for high oilseed yield since it is an oil-type crop, while pak choi was selected for high vegetative mass. The increased seedling vigor of yellow sarson might then be a result of the seeds containing more nutrients (Ambika et al., 2014). Susko and Lovett-Doust (2000) reported a positive effect of seed mass (weight) on higher and faster seed germination and seedling growth in *Alliaria petiolata* (a *Brassicaceae*) and Khan et al. (2012) also observed a positive effect of higher seed weight and larger seed size on seedling vigor traits in tomato. However, during seedling growth yellow sarson was more severely affected by salt stress than pak choi, with more strongly reduced shoot and root length and biomass under salt stress (Table 2; Figure 1; Supplementary Figure S2). One possible explanation of this result could be the thinner seed coat in yellow-seeded genotypes than that of brown/black-seeded genotypes (Xiao et al., 2012). Very often, seed color and the thickness of the seed coat are correlated. However, it should be stressed that seed color and the quantity of the most important fiber compound, acid detergent lignin (ADL), are not strictly linked (Liu et al., 2013). In *B. napus*, the lignin biosynthesis gene CCR1 explained a major QTL on A09 for acid detergent lignin content, which defines seed coat thickness. For a strong seed color QTL, the causal gene, the *bHLH* transcription factor *BrTT8*, was cloned and its role in seed color was functionally validated in *B. rapa* (Li et al., 2012) and in *B. juncea* (Padmaja et al., 2014). The thickness of the seed coat, composed of lignin fibers, as well as its color, composed of pro-anthocyanin (an antioxidant), and antioxidants such as anthocyanin and flavonoids are reported to protect germination under salt stress (Umnajkitikorn et al., 2013) and can protect embryos against biotic and abiotic stresses (reviewed by Boesewinkel and Bouman, 1995).

### Phenotypic variation, correlation and heritabilities of the traits

The variability of root and shoot length in this population increases over the growing days under both conditions (Figure 1; Table 2; Supplementary Figure S2); however, the heritability remained similar (Table 3). The transgressive segregation observed in all traits suggests a quantitative and polygenic inheritance of these traits, requiring a QTL mapping approach to characterize the genetics of seed quality and seedling vigor in *B. rapa*. Root and shoot lengths form two separate clusters (negative correlation), over differences in treatments and seed batches from different years (Supplementary Figure S3) suggesting differences in regulation of root and shoot growth. However, low negative to no correlations between root and shoot lengths may suggest independence to a low level of interdependence (Supplementary Figure S3).

Heritability was lower for seedling vigor traits from the 2010 seed batches (0.4–0.6) than for the 2011 batches (0.5–0.7) (Table 3). In 2011, the DH lines were sown in staggered fashion to synchronize flowering as much as possible and to minimize differing environmental influences during seed development. It is likely that this caused the higher heritabilities in 2011 than in 2010. Higher heritabilities generally resulted in stronger QTLs, illustrated by the fact that most of the QTLs for the 2011 seed batch had higher explained variance (range: 7.1–24.3%; mode value: 14.2%) rather than for the 2010 seed batch (range: 7.8–22.6%; mode value: 11.1%; Supplementary Table S4).

### Major QTL hotspots for seed germination and seedling vigor

We identified eight major hotspots for seed germination and seedling vigor related traits on A02, A03, A05, A08, and A09 (Figure 2A). QTLs for germination were found on hotspot regions on A02 (*Co-QTL2-1* and *-2*); at these loci, the yellow sarson allele is associated with faster germination onset as well as an increase in the germination speed (Figure 2B). It cannot be excluded that *Co-QTL2-1* and *-2* are in fact a single QTL. Additional QTL regions on A01 (*Co-QTL1-3*) and A10 (*Co-QTL10-1*) were mainly associated with rate of germination, and each parent contributed both positive and negative alleles. At *Co-QTL1-3* the yellow sarson allele was associated with higher rate of germination (lower T50), while at *Co-QTL10-1* the pak choi allele associated with higher rate of germination. The fact that across different loci both parents contribute positive alleles is another illustration of the polygenic transgressive nature of the inheritance of these traits.

The proper combinations of QTLs from A01, A02 and A10 can accelerate the onset (T10) and rate or speed (T50) of seed germination in *B. rapa*.

Interestingly, QTLs for flowering time, both in 2010 (peak LOD score 13.4, explained variance 38.0%) and 2011 (LOD 14.8, explained variance 40.9%) mapped to the *Co-QTL2-1* and *-2* regions on A02, which could point to pleiotropy or linkage of QTLs for flowering time and seed germination.

Flowering time regulatory genes have been described to influence agronomic traits such as the number and size of seeds, seedling vigor, biomass, and resistance/tolerance to biotic or abiotic stress (Quijada et al., 2006; Chen et al., 2007; Chiang et al., 2009; Ni et al., 2009; Basunanda et al., 2010; Li et al., 2010; Cartolano et al., 2015), which likely put these genes under selection during crop breeding. The flowering time gene *BrFLC2* maps to this *Co-QTL2-1* region (Xiao et al., 2013) and the expression QTL for the *BrFLC2* gene in leaves of 6 week old plants using the same DH population co-localized with this region (Figure 2B; Table 5). Xiao et al. (2013) identified *BrFLC2* as a major regulator of flowering time and reported the allelic variation between the *BrFLC2* alleles of the two parents yellow sarson and pak choi: a deletion of 56 bp at the exon 4 (12 bp) and intron 4 (44 bp) junction in yellow sarson rendered the gene non-functional; the pak choi allele does not have this deletion. We mapped transcript abundance of *BrFLC2* in developing seeds, resulting in a *cis*-eQTL co-locating with the phenotypic *Co-QTL2-1* and *2-2*.

In the related species *A. thaliana*, Chiang et al. (2009) reported a pleiotropic effect of *FLC* (a homolog of *BrFLC2*) on temperature-dependent germination through additional genes *FT, SOC1* and *AP1* in the flowering time pathway in *A. thaliana*. They also reported the sharing of pathways by flowering time and seed germination, and showed that *FLC* regulates the germination through the abscisic acid catabolic pathway (ABA degradation) and gibberellin biosynthetic pathway in seeds. These observations render *BrFLC2* a candidate gene for the regulation of seed germination; however validation studies are needed to functionally prove its role.

An alternative explanation for the co-localization of *BrFLC2* with QTLs for seed germination could be a major regulatory role of this *FLC2* earliness gene in developmental processes. The possibility of confounding effects of two major loci involved in earliness was reported in *A. thaliana* and potato. In the Landsberg erecta x Cape Verde Islands a RIL population of *A. thaliana*, QTLs for many developmental traits were co-located on the *ERECTA* locus (Stinchcombe et al., 2009). Similarly, in a diploid population of potato, the many QTLs were co-located on the *EARLINESS* locus (Hurtado-Lopez, 2012; Kloosterman et al., 2012). Further, study is needed to deconfound the causal relationships of the *BrFLC2* with seed germination parameters in *B. rapa*.

Many QTLs for germination and seedling vigor under control and salt stress co-localized on *Co-QTL3-3* on A03, *Co-QTL5-1* and *-2* on A05, and *Co-QTL9-2* and *-3* on A09, while several QTLs for seedling vigor only under control and salt stress co-localized on *Co-QTL8-1* on A08 (Figure 2B). Corroborating the finding that there are high correlations between the time points, we found co-localized QTLs for root and shoot lengths measured repeatedly in time. For most traits, two or more than two QTLs were detected. Several putative QTLs co-localized with significant QTLs for correlated traits. QTLs found at multiple time points increase the reliability of these QTLs. Increasing the power of QTL detection by either enlarging the population size or increasing the precision of phenotyping, possibly, could be used to confirm additional candidate QTLs reported in this study.

### QTLs specific to salt stress conditions

QTLs for several germination traits in both the 2010 and 2011 seed batches under salt stress co-localized on *Co-QTL5-1* and *Co-QTL5-2* on A05 and for both loci the yellow sarson allele has a positive effect on maximum germination potential, and rate of germination, but a negative effect on uniformity under salt stress. On hotspots *Co-QTL5-1* and *-2* on A05, also QTLs for root and shoot lengths and shoot weight under salt stress, and relative salt tolerance and 1000-seed weight were mapped (Figure 2B). Finally, QTL for larger seed size and higher 1000-seed weight also map at these loci on A05, with the yellow sarson alleles contributing to a larger seed size and higher 1000-seed weight, which could be in support of a higher maximum germination and faster germination rate in yellow sarson. As marker density was rather low with on average 7-10 cM distance between two markers, more markers and more recombinants are needed to conclude whether the regions actually represent a single or two closely linked QTLs. QTLs at these two hotspots were in coupling phase for all the traits (with the yellow sarson allele having a favorable effect) supporting that this is a single QTL hotspot.

The *BrFAD2* gene, a key gene responsible for biosynthesis of poly-unsaturated fatty acids, is located inside this *Co-QTL5-2* region. Two eQTLs detected in the developing seed transcriptome were mapped for *BrFAD2*: a *cis*-eQTL across the region of *Co-QTL5-1* and *-2* on A05 and a *trans*-eQTL co-locating with *Co-QTL9-2* on A09 (Figures 2, 4). This QTL region on A05 is the major locus with QTLs for seed germination and seedling vigor under salt stress (Figure 2B), suggesting a role of *BrFAD2* in regulating germination and seedling vigor under salt stress. Besides the role of *FAD2* gene in fatty acid desaturation, Wang et al. (2010) reported that the up-regulation of the *FAD2* gene enhanced seed germination and hypocotyl length in their study on *FAD2*-transgenic and non-transgenic lines of the closely related species *B. napus*. In another study on the comparison of a *fad2* mutant of *A. thaliana* with the wild type, a functional role of *FAD2* was reported in increasing salt tolerance during seed germination and early seedling growth (Zhang et al., 2012). *FAD2* extrudes Na^+^ out of the cell and compartmentalizes it into the vacuolar membrane using Na^+^/H^+^ antiporters (NHXs) and thus maintains ion homeostasis. High homology of coding sequences (86%) was found among the homologs of a *FAD2* gene in *A. thaliana, B. rapa* and *B. napus*. Thus, our results in *B. rapa* are in good agreement with the findings on the roles of *FAD2* in *A. thaliana* and *B. napus* and make the *BrFAD2* gene a candidate gene in *B. rapa for* improved seed germination and early seedling vigor under salt stress. For confirmation that FAD2 has a role in salt tolerance in early growth stages, sequencing of the alleles in both parental lines and functional validation of FAD2 in transgenic *B. rapa* is needed.

### Epistatic interaction between QTL hotspots on A02 (Co-QTL2-1 and -2) and on A05 (Co-QTL5-1 and 5-2)

Epistatic interactions between individual genes are prevalent to account for variation in quantitative traits (Jannink and Jansen, 2001). For seed germination and seedling vigor related traits, several studies reported epistatic interactions between genes in *Arabidopsis* (Galpaz and Reymond, 2010; Bouteillé et al., 2012), tomato (Kazmi et al., 2012; Khan et al., 2012), rice (Wang et al., 2012), *B. napus* (Yang et al., 2012) as well as other crops. Interactions with other QTL regions were observed for *Co-QTL2-2, Co-QTL6-2*, and *Co-QTL10-1* indicating that not only main effects of these QTLs but also their epistatic interactions are important for fitness traits like seed germination and seedling vigor (Figure 3). The *Co-QTL2-2* locus showed clear interactions with *Co-QTL5-1* and*-2*, which likely represent a single QTL hotspot, with explained phenotypic variation up to 17.6% of total phenotypic variation. This suggests that besides main effects, also epistatic interactions play a role in the genetic regulatory network of seed germination and seedling vigor in *B. rapa*. A further understanding of interactions with other QTL regions will help to explore the complex genetic architecture of seed germination and seedling vigor in *B. rapa*.

## Author contributions

RB designed and conducted the experiments, analyzed data and wrote the manuscript. AD and DT performed phenotyping, DX carried real time gene expression experiments, SM was involved in marker development, JB provided technical support, RV read the paper, SG supported phenotyping and reviewed the manuscript, GB and CM designed and supervised the experiments and reviewed the manuscript. All authors have read and approved the final manuscript.

### Conflict of interest statement

The authors declare that the research was conducted in the absence of any commercial or financial relationships that could be construed as a potential conflict of interest.

## Supplementary material

Supplementary Figure S1 **Heatmap of Pearson correlation coefficients and hierarchical cluster analysis of seed germination parameters under non-stress (control) and 50 mM NaCl salt stress conditions**. The color bar on the left side indicates the two different seed batches: 2010 and 2011, and the color bars on the top the two different treatments: non-stress (control) and 50 mM NaCl salt. The gradient from red to blue color indicates the degree of positive or negative correlation, respectively.

Supplementary Figure S2 **Box plot showing the distribution of root length (RL) and shoot length (SL) at different days after germination (DAG) under non-stress (control) and salt stress (50 mM NaCl) conditions for the 2010 seed batch**. The shaded color of the boxes indicates the treatments: white for non-stress and gray for salt stress. The y-axis indicates root- and shoot length (in cm). The x-axis label is the combination of RL at 3, 5, 7 and 9 DAG or SL at 3, and 5 DAG under non-stress and salt stress conditions of the 2010 seed batch.

Supplementary Figure S3 **Heatmap of Pearson correlation coefficients and hierarchical cluster analysis of root length (RL) and shoot length (SL) measured at 3, 5, 7, and 9 days after germination (DAG) under non-stress (control) and 50 mM NaCl salt stress conditions**. The color bar on the left side indicates the two different seed batches: 2010 and 2011, the color bars on the top the two different treatments: non-stress (control) and 50 mM NaCl salt. The gradient from red to blue color indicates the degree of positive or negative correlation, respectively.

Supplementary Table S1 **List of traits related to seed weight, seed germination, seedling growth, seedling weight, and salt tolerance parameters with their codes in combination with treatment, DAG (days after germination) and year of the seed batch**.

Supplementary Table S2 **An integrated linkage map (a slightly modified version of Xiao et al. (2013) with map positions of 435 molecular markers on 10 linkage groups for this ***B. rapa*** doubled haploid population**.

Supplementary Table S3 **Summary of significant QTLs identified for seed germination parameters and seed weight (1000-seed weight) in the ***B. rapa*** DH population from a cross of yellow sarson (YS143) and pak choi (PC175)**.

Supplementary Table S4 **Summary of significant QTLs identified for root length (RL), shoot length (SL), root weight, and shoot weight in the *B. rapa* DH population from a cross of yellow sarson (YS143) and pak choi (PC175)**.

Supplementary Table S5 **Summary of significant QTLs identified for salt tolerance parameters in the ***B. rapa*** DH population from a cross of yellow sarson (YS143) and pak choi (PC175)**.

Supplementary Table S6 **QTL profiles with LOD score values for all the markers as well as pseudo-markers at intermediate map positions for all the traits shown in Figure 2B**. A positive LOD score indicates that the YS143 allele increases the trait value, a negative LOD score that the PC175 allele increases the trait values. Pseudomarkers generated by the interval mapping procedure in MapQTL are denoted by “Z” followed by numeric values.
