# Supplementary figures and images for: Quantitative Trait Locus Analysis of Seed Germination and Seedling Vigor in Brassica rapa Reveals QTL Hotspots and Epistatic Interactions
Source: Front Plant Sci. 2015 Dec 1;6:1032. doi: 10.3389/fpls.2015.01032 (PMC4664704; doi:10.3389/fpls.2015.01032)

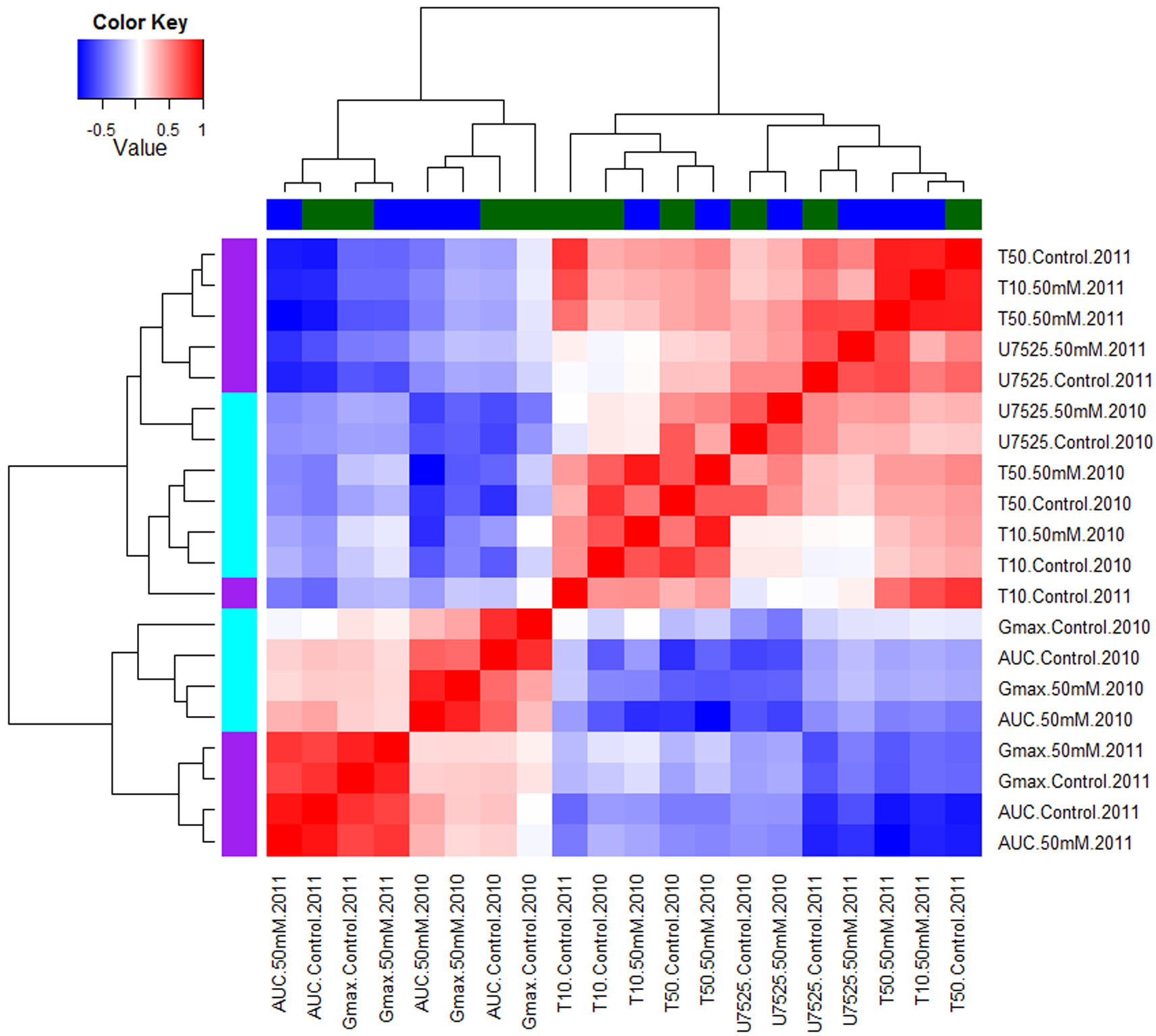

Supplement: Supplementary Figure S1 — Heatmap of Pearson correlation coefficients and hierarchical cluster analysis of seed germination parameters under non-stress (control) and 50 mM NaCl salt stress conditions. The color bar on the left side indicates the two different seed batches: 2010 and 2011, and the color bars on the top the two different treatments: non-stress (control) and 50 mM NaCl salt. The gradient from red to blue color indicates the degree of positive or negative correlation, respectively. [file Image1.TIFF]

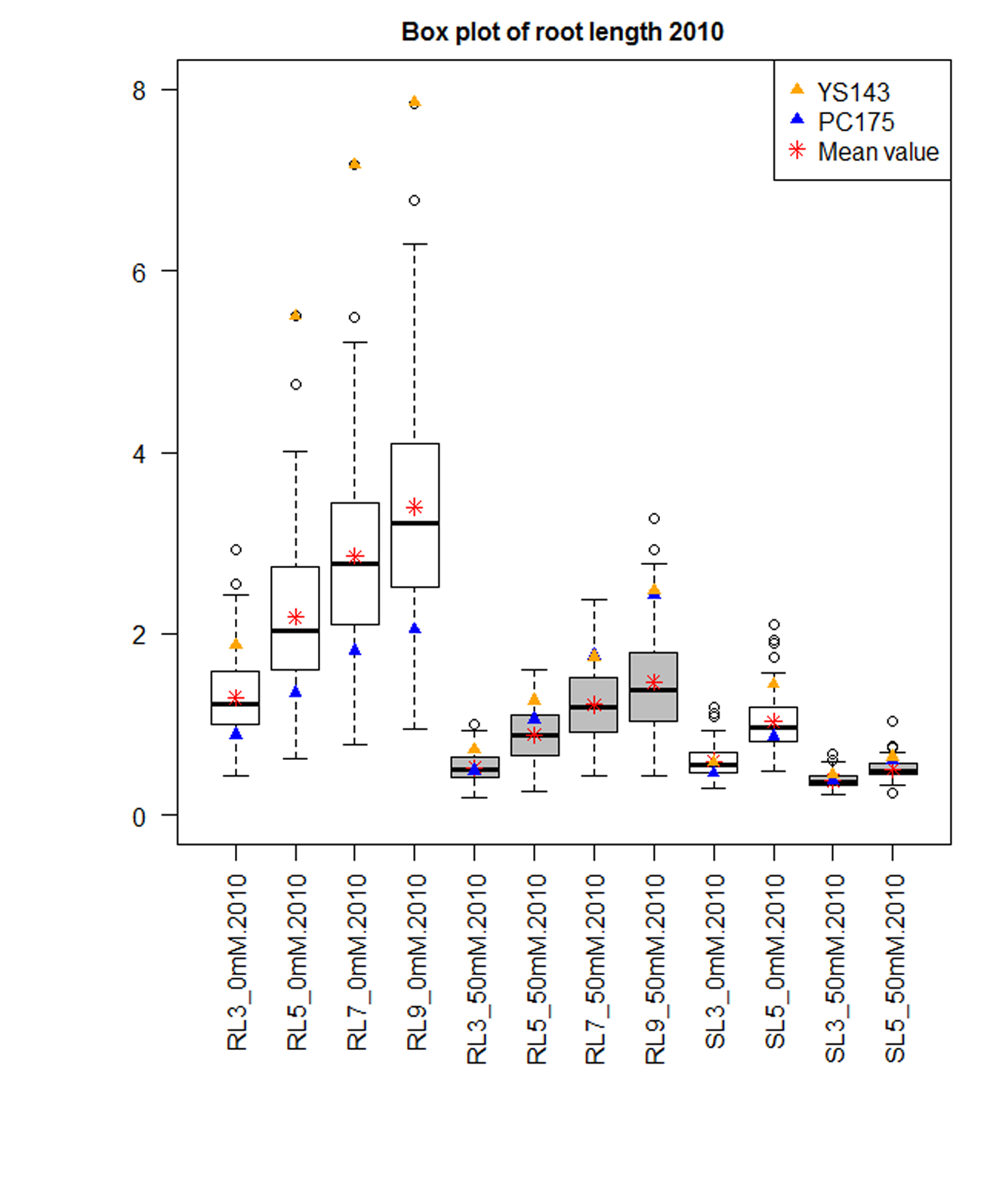

Supplement: Supplementary Figure S2 — Box plot showing the distribution of root length (RL) and shoot length (SL) at different days after germination (DAG) under non-stress (control) and salt stress (50 mM NaCl) conditions for the 2010 seed batch. The shaded color of the boxes indicates the treatments: white for non-stress and gray for salt stress. The y-axis indicates root- and shoot length (in cm). The x-axis label is the combination of RL at 3, 5, 7 and 9 DAG or SL at 3, and 5 DAG under non-stress and salt stress conditions of the 2010 seed batch. [file Image2.TIFF]
